# Teleophthalmology: A Model for Eye Care Delivery in Rural and Underserved Areas of India

**DOI:** 10.1155/2011/683267

**Published:** 2011-07-17

**Authors:** Vijayaraghavan Prathiba, Mohan Rema

**Affiliations:** Dr. Mohan's Diabetes Specialities Centre and Madras Diabetes Research Foundation, 6 Conran Smith Road Gopalapuram, Chennai 600086, India

## Abstract

*Objectives.* To describe the application of teleophthalmology in rural and underserved areas of India. *Study Design.* This paper describes the major teleophthalmology projects in India and its benefits. *Results.* Teleophthalmology is the use of telecommunication for electronic transfer of health-related data from rural and underserved areas of India to specialities in urban cities. The MDRF/WDF Rural Diabetes Project has proved to be very beneficial for improvement of quality health care in Tamilnadu and can be replicated at the national level. This community outreach programme using telemedicine facilities has increased awareness of eye diseases, improved access to specialized health care, helped in local community empowerment, and provided employment opportunities. Early detection of sight threatening disorders by teleophthalmology and prompt treatment can help decrease visual impairment. *Conclusion.* Teleophthalmology can be a very effective model for improving eye care delivery system in rural and underserved areas of India.

## 1. Introduction

Telemedicine is the integration of electronic information and medical technology by which people in remote and underserved areas can get access to specialized expert health care. Telemedicine is a rapidly developing field thanks to application of clinical medicine by telephone, the internet, or other networks for the purpose of consultations and on occasion, carrying out examinations or medical procedures [1]. This may be as simple as two health professionals discussing a case over the telephone, or as complex as using satellite technology and video-conferencing equipment to conduct a real-time consultation between medical specialists in two different countries. Telemedicine has special significance to India considering its vast geographical spread and predominant rural population where medical care is neither available nor accessible [2]. While 72% of India's 1.2 billion people live in rural areas, over 70% of the doctors practice in urban areas. This article reviews the present status of the application of telemedicine in the field of ophthalmology (teleophthalmology) and how this technology may be used to provide ophthalmologic services to rural, underserved, and impoverished parts of India.

## 2. Examples of Teleophthalmology Projects in India

**Figure 1:**
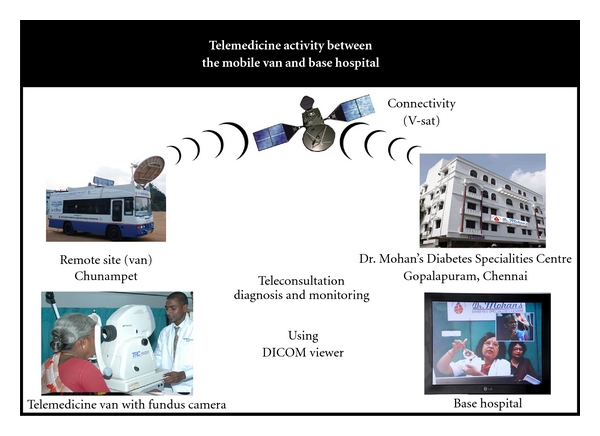


One of the important ongoing projects in telemedicine started by Madras Diabetic Research Foundation (MDRF), Chennai, in collaboration with the World Diabetes Foundation (WDF), is the MDRF/WDF Rural India Diabetes Prevention Project. This rural community outreach programme serves a cluster of 42 villages (in and round Chunmpet village) in Kancheepuram District, Tamilnadu, India. Screening is carried out in Chunampet district for diabetes and its complications especially diabetic eye diseases by using a mobile telemedicine van with satellite connectivity. The telemedicine van is equipped with a digital retinal camera by which retinal imaging is performed by eye technicians who are unemployed youth recruited from the local area and trained at our centre. All quadrants of the retina are imaged by the eye technician. Nearly 2000 patients with diabetes have undergone retinal color photography to date. These images are then transmitted via very small aperture terminal (VSAT) satellite connectivity provided by the Indian Space Research Organization (ISRO) to our base hospital in Chennai. By video conferencing, the ophthalmologist in the base hospital interacts with the patients in rural and underserved areas.  Figure 1 presents how the teleophthalmology is done at our centre. Those patients needing further treatment are brought to Chennai, where laser photocoagulation/cataract surgery is performed free of cost. One of the main advantages of teleophthalmology is early detection of sight-threatening changes which can be treated. The patients treated here serve as health ambassadors to further disseminate information about benefits of teleophthalmology to surrounding villages and also help remove superstitious beliefs. Screening for other diabetes-related complications like nephropathy (by measuring proteinuria/microalbuminuria), neuropathy (by biothesiometry, and monofilament) and coronary artery disease (by electrocardiogram) is also done in the telemedicine van. The recent establishment of the Sai Rural Diabetes Specialities Centre with all latest facilities in Chunampet has made followup easier and also allows the telemedicine van to increase its area of coverage. Employment opportunities have been provided to many of the local youth, thus providing a source of livelihood to the villages. This successful rural health care model can also be implemented in other parts of our country. The Chunampet Rural Diabetes project has been recognized as a model for delivering diabetes care to rural areas in developing countries [3]. The telemedicine facility is also being used by us to conduct CME programs for other hospitals in India. 

The teleophthalmology project conducted by Sankara Nethralaya Medical Research Foundation at Chennai uses a customized mobile van with an in-built ophthalmic examination facility having satellite connectivity provided by ISRO, along with a social worker and an optometrist [4]. Patients undergo a preliminary screening for ophthalmic diseases at camps organized at these villages. Photographs are taken using a digital camera having a resolution of 3.2 mega pixel (Canon USA Inc.). After pupillary dilatation, a single 45° digital fundus photograph centered midway between the center of the macula and the disc is taken with Topcon TRC NW 100 nonmydriatic camera. Real-time interaction by the ophthalmologist with the examining optometrist, as well as the patient, is then established using the videoconferencing system. 

Another example of application of technology in rural heath care is the Aravind Teleophthalmology Network. A mobile eye-screening van fitted with a satellite has been specially designed to screen the diabetic patients in the camps, hospitals, and clinics of diabetologists. Up to July 2006 [5], 74 screening camps have been conducted and 20,080 patients have been screened in the van. The Aravind Comprehensive Eye Survey Research Group Study [6] showed that the prevalence of diabetic retinopathy in rural South Indian population was 10.5%. Only 6.7% of individuals with diabetic retinopathy had previous eye examinations. 

Based on our experience for screening of diabetic retinopathy, we feel it is ideal to take pictures of all the quadrants of the retina including the macula and optic disc after dilatation of the pupils. When this is not feasible due to resource constraints, or for logistic reasons, it is best to take four fields:macula, nasal to disc, superior temporal, and inferior temporal quadrants using a nonmydriatic digital camera.

To support telemedicine activities within the country, the Department of Information Technology has defined the Standards for Telemedicine Systems, and the Ministry of Health and Family Welfare has constituted the National Telemedicine Task Force [7]. ISRO has coupled its prowess in satellite communication technology with medical science and information technology to project specialty healthcare to the doorsteps of the remote, rural, and underserved populations across the country [8]. Studies carried out between 2000 and 2001 by the Apollo Telemedicine Networking Foundation [9], were instrumental in ISRO including telemedicine as a major thrust area for the country. Indian telemedicine establishments also need periodic evaluation to rationalize the main objective of the technology, that is, patient care, patient satisfaction, and patient opinion, all leading to patient empowerment [10].

The landmark population-based study Chennai Urban Rural Epidemiology Study [11] carried out by the Madras Diabetes Research Foundation showed the prevalence of diabetic retinopathy in urban population to be 17.6%. Since diabetes remains largely underdiagnosed in rural populations, it is likely that the burden owing to diabetic retinopathy and other complications will be enormous in India. Using telemedicine, a collaborative effort by health care providers, nongovernmental organizations (NGOs) and the government can help improve accessibility of rural population to better health care.

## 3. Benefits of Teleophthalmology

Table 1 summarizes the benefits of teleophthalmology applications which include detecting, screening, and diagnosing diabetic retinopathy; anterior segment imaging; glaucoma screening; low vision consultation and telementoring [12]. Teleophthalmology can be performed in realtime, by store-and-forward mode, or by hybrid techniques. In a screening done in rural South Indian population by telehealth facilities [13], diabetic retinopathy was detected in 19% of rural people with diabetes, 73.6% of whom had never undergone an eye examination. In another study [4], 2.8% of patients were detected to have adnexal and orbital diseases by teleophthalmology. Of these patients, surgery was advised in 61.3% patients, medical treatment in 12.8%, and the rest needed further investigations at a tertiary center. In a study done to assess satisfaction levels [14], patients who asked questions during teleconsultation were 2.18-times more likely to be satisfied with teleophthalmology than who did not. Cost-effectiveness analysis of screening for diabetic retinopathy was performed using two models, teleophthalmology model and the base hospital screening model [15]. The telescreening was found to have a considerably lower cost per case in comparison to the base hospital. 

Teleophthalmology could be particularly useful in primary care where the distance to an ophthalmologist can be a significant obstacle to satisfactory diagnosis and treatment [16]. However, there is no data from India on this, and studies are urgently needed in this area.

To conclude, teleophthalmology is an exciting new technology which can help in integration of all urban and rural health care centers in India and improve the quality of medical services in the presently underserved and impoverished sections particularly in remote rural areas of developing countries like India.

## Figures and Tables

**Table 1:** Benefits of teleophthalmology.

(1)	Diagnosis and formulation of treatment plans for diabetic retinopathy
(2)	Visual acuity testing and refraction
(3)	Low vision consultations
(4)	Cataract screening
(5)	Glaucoma screening
(6)	Anterior segment imaging
(7)	Continuing medical education by tele-education
(8)	Transferring of ultrasound, electrooculography and electroretinography images
(9)	Employment facilities to the local unemployed youth
(10)	Can reach remote rural and underserved areas where specialized medical care is not available

## References

[1] Sharma LK, Rajput M (2009). Telemedicine: socio-ethical considerations in the Indian milieu. *The Medico-legal journal*.

[2] Bedi BS (2009). Telemedicine standards: need and Indian initiatives. *Telemedicine and E-Health*.

[3] Patel V, Chatterji S, Chisholm D (2011). India: towards universal health coverage 3–chronic diseases and injuries in India. *Lancet*.

[4] Verma M, Raman R, Mohan RE (2009). Application of tele-ophthalmology in remote diagnosis and management of adnexal and orbital diseases. *Indian Journal of Ophthalmology*.

[5] Bai VT, Murali V, Kim R, Srivatsa SK (2007). Teleophthalmology-based rural eye care in India. *Telemedicine Journal and e-Health*.

[6] Nirmalan PK, Katz J, Robin AL (2004). Prevalence of vitreoretinal disorders in a rural population of Southern India: the aravind comprehensive eye study. *Archives of Ophthalmology*.

[7] Mishra SK, Kapoor L, Singh IP (2009). Telemedicine in India: current scenario and the future. *Telemedicine and E-Health*.

[8] Bhaskaranarayana A, Satyamurthy LS, Remilla MLN (2009). Indian space research organization and telemedicine in India. *Telemedicine and E-Health*.

[9] Ganapathy K, Ravindra A (2009). Telemedicine in India: the apollo story. *Telemedicine and E-Health*.

[10] Bhatia JS, Sharma S Telemedicine endurance-empowering care recipients in Asian Telemedicine setup.

[11] Rema M, Premkumar S, Anitha B, Deepa R, Pradeepa R, Mohan V (2005). Prevalence of diabetic retinopathy in urban India: the Chennai Urban Rural Epidemiology Study (CURES) Eye Study—I. *Investigative Ophthalmology and Visual Science*.

[12] Tang RA, Morales M, Ricur G, Schiffman JS (2005). Telemedicine for eye care. *Journal of Telemedicine and Telecare*.

[13] Raman R, Mahajan S, Padmaja RK, Agarwal S, Gnanamoorthy P, Paul PG (2005). Tele-health program for diabetic retinopathy in Rural South India: a pilot study. *E-Health International*.

[14] Paul PG, Raman R, Rani PK, Deshmukh H, Sharma T (2006). Patient satisfaction levels during teleophthalmology consultation in rural south India. *Telemedicine Journal and e-Health*.

[15] Sudhir RR, Frick KD, Raman R, Padmaja RK, Murali V, Sharma T (2005). Mobile teleophthalmology: a cost effective screening tool for diabetic retinopathy in rural South India. *E-Health International*.

[16] Blomdahl S, Marén N, Lof R (2001). Tele-ophthalmology for the treatment in primary care of disorders in the anterior part of the eye. *Journal of Telemedicine and Telecare*.

